# Psychosocial vulnerability of people working in informal gold mines in French Guiana: a multicentric cross-sectional survey in 2024–2025

**DOI:** 10.3389/fpubh.2026.1894124

**Published:** 2026-09-11

**Authors:** Maylis Douine, Yann Lambert, Teddy Bardon, Lorraine Plessis, Marion Petit-Sinturel, Raphaëlle Le Querriou, Carlotta Carboni, Amanda Figueira Da Silva, Josiane Nogueira Muller, Célia Basurko, Astrid Van-Melle, Antoine Adenis, Mathieu Nacher, Stephen Vreden, Martha Suárez-Mutis, Alice Sanna

**Affiliations:** 1 Centre d'Investigation Clinique Guyane Amazonie, Inserm 1424, Département recherche-Innovation-Santé Publique, Centre Hospitalier Universitaire de Guyane – Site Cayenne, Cayenne, French Guiana; 2 UMR UA17 Santé Des Populations en Amazonie, Cayenne, French Guiana; 3 Laboratory of Parasitic Diseases, Institute Oswaldo Cruz/Fiocruz, Rio de Janeiro, Brazil; 4 Foundation for the Advancement of Scientific Research in Suriname (SWOS), Paramaribo, Suriname

**Keywords:** access to care, food insecurity, gold miners, mental health, social vulnerability, violence

## Abstract

**Introduction:**

In French Guiana, workers involved in illegal gold mining—mainly migrants from Brazil's poorest states—constitute a highly vulnerable population. Living and working without legal status in remote Amazonian areas, they face limited access to essential health and social services and unstable conditions (1, 2). This study aimed to describe psychosocial vulnerability in this hard-to-reach population, identify associated factors, and explore vulnerability profiles.

**Methods:**

A cross-sectional descriptive survey was conducted among adults engaged in illegal gold mining in French Guiana (3). Participants were enrolled at logistical bases along the borders with Suriname and Brazil. After informed consent, trained field investigators administered a standardized questionnaire covering sociodemographic characteristics, mining activities, mental health, violence exposure, food insecurity, perceived safety, quality of life, and healthcare access. Data were analyzed using descriptive statistics, validated scores, and cluster analysis to identify vulnerability profiles.

**Results:**

Between December 2024 and April 2025, 480 participants were enrolled, 95.4% of whom were Brazilian. Over half (55.7%) reported medium or poor health, and 43.9% lived more than a day's travel (by boat or on foot) from the nearest health facility. Social vulnerability was substantial: 16.7% reported no social support, 43.7% felt unsafe, and 29.8% had been exposed to violence. Food insecurity was frequent, with 20.5% experiencing moderate hunger and 0.4% severe hunger. Overall, 21.9% screened at elevated mental-health vulnerability. Food insecurity was independently associated with inclusion in Brazil, financial difficulties, lack of social support, and social isolation. Mental-health vulnerability was associated with female sex, social isolation, and feeling unsafe at mining sites.

**Conclusion:**

Brazilian migrants involved in illegal gold mining in French Guiana face substantial multidimensional vulnerabilities. The findings highlight cumulative vulnerabilities and support the need to consider targeted harm-reduction and coordinated cross-border public-health strategies to promote health equity.

Trial registration number: NCT05540470.

## 1 Introduction

In health sciences, vulnerability refers to factors that increase individuals' sensitivity to harm or diminish their capacity to cope with risks, without necessarily acting as direct causal agents of disease. Willen and colleagues conceptualize vulnerability as “the exposure to a set of conditions that render individuals and communities more susceptible to disease or disability” (1, 2). Vulnerable populations are therefore social groups subject to structural constraints and resource scarcity, resulting in disproportionately high risks of morbidity and premature mortality. Understanding the multiple dimensions of vulnerability is essential for designing effective public health interventions, particularly for marginalized and hard-to-reach populations.

Informal gold mining is a long-standing activity worldwide and is particularly prevalent in the Guiana Shield, where thousands of individuals operate in remote forested areas under severe environmental, social, and legal hardships (3, 4). In French Guiana, an overseas department of France within this region, an estimated 10,000 people – predominantly migrant workers from Brazil – are engaged in illegal mining activities. Existing research has largely focused on infectious diseases among this population, particularly malaria and leishmaniasis, as well as heavy metal exposure such as mercury and lead (3, 5–8). A recent study by our team on social determinants of health documented extremely precarious living conditions among clandestine miners, including limited resources, lack of potable water, proximity to dangerous wildlife, frequent occupational accidents, treatment interruptions for chronic diseases, and delays in accessing medical services (9). Furthermore, illegal gold miners are also frequently exposed to violence in different regions of the world (10–13). These factors collectively make informal gold miners highly vulnerable.

Their living conditions and their vulnerability can profoundly affect their mental health. The World Health Organization defines mental health as “a state of wellbeing that enables individuals to realize their abilities, cope with normal stresses of life, and contribute to their community.” The United Nations included mental health among the Sustainable Development Goals (SDG 3.4) (14). This underscores the urgent need to address psychosocial risks across all population segments, including hard-to-reach groups such as informal miners. Evidence from other marginalized contexts consistently demonstrates that chronic insecurity, social marginalization, exposure to violence, and material deprivation are strong predictors of psychological distress and poor mental health outcomes (15).

In French Guiana, informal mining takes place in remote forested areas that are regularly subjected to police interventions – called Opérations Harpie – aimed at curbing illegal gold extraction. While these operations pursue important environmental protection and security objectives, they exacerbate miners' precarity by disrupting access to food supplies, hampering mobility, and further limiting healthcare access. These cumulative pressures can severely undermine individuals' capacity to cope with daily stressors and amplify broader psychosocial vulnerability.

Despite these high-risk conditions, the psychosocial vulnerabilities of this population remain poorly documented. This survey was therefore designed to address this gap. The objectives were threefold: (i) to describe the psychosocial vulnerability of individuals working in informal mining sites in French Guiana; (ii) to identify factors associated with mental health status and food insecurity; and (iii) to explore distinct vulnerability profiles within this hard-to-reach population.

## 2 Methods

### 2.1 Study population and sampling size

The study population comprised individuals engaged in informal gold mining activities in French Guiana. Eligibility criteria included being present on a mining site within the 7 days preceding enrollment, providing written informed consent, and being aged 18 years or older. The required sample size was determined according to the primary objective of the survey, estimating malaria prevalence. Assuming a 2% baseline prevalence and a 75% reduction after 2 years, 860 participants per survey were required (α = 0.05, power = 80%) (16).

### 2.2 Study design

This research consisted of a cross-sectional descriptive survey using data collected through a structured questionnaire. It was conducted as part of the interventional Curema project, designed to assess an innovative strategy for malaria elimination in this vulnerable population (16). Participant enrollment occurred between December 2024 and April 2025.

Study participants were identified in logistical staging areas, where gold miners routinely stop for trading gold, purchase supplies, rest, or visit relatives. These hubs are situated along the Maroni River at the border with Suriname and in the city of Paramaribo, and along the Oyapock River, at the border with Brazil. Recruitment was undertaken by a multidisciplinary team composed of a health mediator and a nurse. In the team at the Surinamese border, a physician was also included. After confirming eligibility and ensuring that the participant had not previously taken part in the same study, the written informed consent form was obtained.

The recruitment process combined chance encounters with a snowball sampling approach, which is particularly suited to engaging hidden or hard-to-reach populations.

### 2.3 Data collection

The questionnaire included variables related to socioeconomic status, gold mining activity, lifestyle (including smartphone ownership and internet connection), mental health, exposure to violences, food insecurity, quality of life, chronic diseases and access to care (Supplementary. Material 1).

The questionnaire was adapted from previous surveys conducted among gold miners and used different validated scores or scales. Items assessing social support and perceived isolation were adapted from the VESPA questionnaire developed for people living with HIV (17). Food insecurity was assessed at the participant level through the Household Hunger Scale (HHS), a validated score including three questions (18). Depression symptoms were assessed through the two questions from Patient Health Questionnaire depression module (PHQ-2) (19). The notion of trauma was explored through a highly subjective question:

“*Do you feel you have experienced one or more traumatic events in your life?”*

Trauma was thus considered as a perceived experience rather than through a predefined list of potentially traumatic events.

In addition to the quantitative questionnaire, two open-ended questions were asked:

“*What were the reasons that led you to start gold mining?”* and “*What are the reasons why you continue in this activity?”*

The questionnaire was pre-tested on five individuals from the target population to assess both comprehension and acceptability of the questions. Some questions related to secondary objectives had to be shortened or clarified to improve understanding and reduce administration time. Thus, items derived from the PHQ-2 were presented in a binary response format (yes/no), instead of the original frequency scale. It was administered by two health mediators specifically trained for the survey, in the participant's native language (primarily Portuguese).

### 2.4 Statistical analysis

Descriptive statistics were used to summarize the data. Qualitative variables were presented as frequencies and percentages, while quantitative variables were expressed as medians with interquartile ranges. Missing data were explicitly reported in the tables. “Other gender” was classified as “female” for the analysis of psychosocial vulnerability. Work activities in mining camps were classified as mobile (itinerant vendors, canoe drivers, transporters) or non-mobile (gold miners, cooks, machine operators).

The three items of the HHS were coded as 0 = never experienced, 1 = rarely/sometimes, 2 = often. Then the HHS score was calculated: 0–1 little to no hunger in the household; 2–3 moderate hunger in the household; 4–6 severe hunger (18). This score was then categorized as food secure (HHS = 0–1) or food insecure (HHS = 2–6) for the analysis of associated factors.

A study-specific mental-health vulnerability score was constructed using the two PHQ-2 items administered in a binary format (yes/no), together with two additional questions assessing persistent fatigue and previous psychiatric or psychological care: “During the past 2 weeks, did you feel tired almost all the time, without energy?” and “Have you ever been followed or hospitalized by a psychiatrist or psychologist for mental health problems?”. This score was used as an exploratory proxy for psychological distress. One point was assigned for each positive response, yielding a score ranging from 0 to 4. Because the PHQ-2 was not administered using its original frequency response scale, its validated scoring and cut-off were not applied. The score was dichotomized as 0–1 vs. ≥2 using a pragmatic, study-specific threshold corresponding to the presence of at least two mental-health-related indicators. This threshold was not intended to represent a clinically validated cut-off or to diagnose a mental disorder. Because the threshold was exploratory, sensitivity analyses were performed using alternative definitions of the outcome.

Data were analyzed using Stata 19.0 (StataCorp LLC, College Station, TX, USA).

#### 2.4.1 Descriptive content analysis of open-ended questions

We conducted a descriptive content analysis of the two open-ended responses exploring motivations for entering and remaining in artisanal gold mining. Responses were manually coded into key themes (economic necessity, rapid income, improved living conditions, family obligations, habituation). Approximate frequency counts and illustrative quotes were used to enrich interpretation. Multiple themes could be assigned to a single response when appropriate. The coding was performed in Portuguese by one researcher (MD). Approximate frequency counts and illustrative quotes were used to enrich the interpretation. Responses provided in Portuguese were translated into English by MD and AS for the manuscript. Given the brief nature of the responses, this analysis was intended to identify broad socioeconomic and psychosocial patterns rather than to constitute an in-depth qualitative or thematic analysis.

#### 2.4.2 Multivariate analysis of factors associated with food insecurity and mental health

Modified Poisson regression with a robust variance estimator was applied to identify factors associated with food insecurity (HHS 0–1 vs. 2–6) and mental health vulnerability. This approach was used to directly estimate prevalence ratios (PRs) and 95% confidence intervals (CIs). Variables with a *p*-value < 0.20 in univariable analyses were considered for inclusion in the multivariable model. Age, sex, and recruitment site were retained *a priori* in all adjusted models, regardless of statistical significance, because they were considered important design-related or potential confounding variables. A stepwise selection procedure was then applied to the other candidate variables, using a retention threshold of *p* < 0.05. Missing data were rare ( ≤ 12 participants out of 480) and were handled using complete-case analysis. Potential multicollinearity and interactions were assessed.

#### 2.4.3 Multiple correspondence analysis and clustering

Multiple correspondence analysis (MCA) was performed on key vulnerability indicators to identify underlying patterns of vulnerability using complete-case analysis (20). Several MCA specifications were explored by adding or removing vulnerability indicators. The final specification was selected based on the discrimination of the variables on the resulting factorial map. Individual coordinates on the first two MCA dimensions were subsequently used for k-means clustering. Solutions with two and three clusters were compared, and the three-cluster solution was retained based on cluster interpretability and the distinctiveness of the resulting vulnerability profiles. The clusters were then characterized by comparing the distribution of the original vulnerability indicators across groups. Because these analyses were exploratory, the clusters were considered descriptive and hypothesis-generating rather than definitive population subgroups.

### 2.5 Ethical considerations

Written informed consent was obtained from all participants after providing information in their native language regarding the primary objective of the study (malaria) and the secondary objectives (assessment of psychosocial vulnerability).

As part of the Curema project, the study received approval from the Surinamese Ministry of Public Health (N°CMWO005) and from the Brazilian National Research Ethics Commission (N°5.507.241). All data were anonymized and registered in compliance with the General Data Protection Regulation. The study was carried out in accordance with the principles of the Declaration of Helsinki.

### 2.6 Patient and public involvement

The study population was involved in the development of the questionnaire (with the definition of meaningful questions and appropriate wording), and pretesting of the questionnaire (comprehension, acceptability, duration). Recruitment of participants was done by opportunistic meeting and then by snowball sampling, thus involving the participants themselves. The first part of the questionnaire was administered by a health mediator, who is a pair issued from the target community.

### 2.7 Funding

This work was supported by the European Interreg Amazon Cooperation Program (N° Presage 8754) and the French Guiana Regional Health Agency. The funders had no role in study design, data collection and analysis, or in the decision to submit the manuscript for publication.

## 3 Results

### 3.1 Study population

During the 5-month study period, 489 individuals were enrolled. Approximately 40 refusals were documented, although refusal data were not recorded exhaustively at all sites because of periods of high patient flow and the time required for the informed-consent process. Lack of time was the main reported reason for refusal. Of these 489 enrolled, two informed consent forms were lost, and seven questionnaires could not be analyzed due to data loss related to technical issues; thus, 480 participants with complete questionnaires were included in the analysis. The male-to-female sex ratio was 3.16, and the median age was 42 years [interquartile range (IQR): 34–52]. One participant reported “other gender”. Although 95.4% of them were born in Brazil, 289 participants were recruited at logistical staging areas at the Suriname border and 191 at the Brazil border. Nearly two-thirds (63.3%) had no formal education or only primary school level. Participants had been engaging in gold mining for a median of 13 years [IQR: 5–20].

### 3.2 Health condition

Self-reported health status was rated as good by 46.5% of participants and as medium by 46.9%, while 5.8% considered their health to be poor (0.8% doesn't know or doesn't want to answer (dnk/dnwta)). Only a minority reported chronic diseases (8.1%, *n* = 39), of whom two-thirds (66.7%) declared having taken chronic treatment during the past week. Regarding access to healthcare, 11.5% of respondents reported having foregone medical care when needed. The main reasons for not accessing the health system were waiting times before appointments (*n* = 23), long distance to facilities (*n* = 10), and financial difficulties (*n* = 7).

### 3.3 Socio-economic situation in the mine

For 43.9% of participants (207/472), the distance between the mining site and the resting place where recruitment took place was more than 1 day, and could extend up to 10 days. The median travel cost was estimated at €100 [IQR: 50–272] (equivalent in July 2025, as transactions are most often made in grams of gold). Costs varied according to travel time, with a median of €68 [IQR: 50–136] for trips of up to 1 day, and €167 [IQR: 68–340] for trips exceeding 1 day.

Regarding financial situation, about half of the participants (49.8%) reported being comfortable, while 24.8% considered their situation moderately difficult and 17.5% described it as difficult. A further 7.9% did not know or preferred not to answer.

A total of 47.3% reported having experienced a police operation during the previous year. Among these 227 persons, 29.0% declared having faced more than 10 operations, while 47.1% reported fewer than 5. The most recent police operation occurred with a median of 2 weeks prior to the interview [IQR = 0.7–12.8].

One third of participants (159/480, 33.1%) reported increased mobility difficulties between mining sites or when trying to leave them during the past 2 years compared to before. The main reasons mentioned were the presence of police enforcement operation (*n* = 117), lack of financial resources (*n* = 18), and drought making rivers, the main means of transportation in the Amazon, difficult to navigate (*n* = 17).

#### 3.3.1 Reasons for starting gold mining activity

The reasons given by the 475 respondants (3 dnk) for working in the gold mines revolve mainly around three major themes: economic necessity, the search for a better life, and the lack of professional alternatives (“falta de trabalho” – lack of work; “única opção” – only option). Family and social pressure (“influências de amigos” – influence of friends; “com a minha família” – with my family) also appear, as well as a more existential register mixing curiosity, adventure, and the dream of getting rich (“curiosidade” – curiosity; “sonho de rica” – dream of being rich). Finally, the lack of schooling (“não tem estudo” – no education; “analfabeto” – illiterate) is often presented as a barrier to other opportunities (Table 1).

**Table 1:** Descriptive content analysis of factors associated with entering gold mining and reasons for remaining in gold mining.

Descriptive content analysis of factors associated with entering gold mining		
Theme	Occurrences	Illustrative quotations
Necessity	80	*necessidade* (necessity); *falta de trabalho na cidade* (lack of work in the city); ú*nica opção* (only option); *não tem estudo* (no education); *necessidade financeira* (financial need)
Money	65	*dinheiro* (money); *dinhero* (money, misspelling); *ganha dinheiro rápido* (earn quick money); *forma mais rápida pra ganhar dinheiro* (the fastest way to earn money); *dinheiro bom* (good money)
Improvement of life	55	*procura a melhora* (looking for improvement); *melhorar de vida* (improve life); *busca de uma vida melhor* (search for a better life); *mudar de vida* (change life); *melhor opção* (better option)
Family and network	20	*com a minha família* (with my family); *criar meus irmãos* (raise my siblings); *por causa do marido* (because of husband); *influência de amigos* (influence of friends); *família dentro do garimpo* (family inside the mining site)
Curiosity/dream	10	*curiosidade* (curiosity); *aventura* (adventure); *sonho de rica* (dream of being rich); *paixão* (passion); *busca de um sonho* (pursuit of a dream)
Necessity/economic survival	120	*necessidade* (necessity); *falta de emprego* (lack of employment); ú*nica opção* (only option); *problema financeira* (financial problem); *para ganhar dinheiro para uma vida melhor* (to earn money for a better life)
Money/quick earnings	100	*dinheiro rápido* (quick money); *ganhar mais* (earn more); *dinheiro bom* (good money); *forma rápida de ganhar dinheiro* (quick way to earn money); *ganha mais que no Brasil* (earn more than in Brazil)
Improvement of living conditions	70	*melhorar de vida* (improve life); *procura a melhora* (looking for improvement); *busca da melhora* (search for improvement); *melhor opção para evoluir* (better option to progress); *construir casa própria* (build one's own house)
Supporting the family	40	*família* (family); *filhos* (children); *manter a família* (maintain the family); *dar o melhor pra família* (give the best to the family); *sustentar a família* (support the family)
Habit/routine/work attraction	25	*acostumado* (accustomed); *gostei da atividade* (I liked the activity); *virou um vício* (it became like an addiction); *não quer voltar pra roça* (doesn't want to return to farming); *aventura* (adventure)

#### 3.3.2 Reasons for continuing gold mining activity

The reasons put forward by the 475 respondants (1 dnk) to explain why they remain in the gold mining activity are organized around five main themes. First, economic necessity and survival are the main drivers: many evoke “necessidade” (necessity), “falta de oportunidades” (lack of opportunities), and “única opção” (only option), reflecting the absence of alternatives to meet their needs. Next, the search for immediate and high income is omnipresent (“dinheiro rápido” – quick money; “ganhar mais” – earn more; “forma rápida de ganhar dinheiro” – quick way to earn money). Added to this is the desire to improve living conditions, whether to “construir uma casa” (build a house), “mudar de vida” (change life), or “procura a melhora” (looking for improvement). Arguments related to family and children are also frequent, with miners highlighting the need to “sustentar a família” (support the family) or “dar o melhor pra família” (give the best to the family). Finally, some testimonies reveal an attachment to the routine of the garimpo, between ingrained habits, enjoyment of the work (“gostei da atividade” – I liked the activity) and sometimes even a “vício”, suggesting that some participants found it difficult to give up mining and perceived the environment as potentially addictive (Table 1).

### 3.4 Quality of life

Regarding current quality of life, 34.4% of participants (95% Confidence Interval (CI): 30.3–38.7) reported it as good, 52.7% (95% CI: 48.3–57.1) as medium, and 12.9% (95% CI: 10.1–16.3) as poor or not known (*N* = 480).

When asked to compare their present life with the period before gold mining, 48.0% (95% CI: 43.5–52.5) considered it better, 17.7% (95% CI: 14.4–21.6) the same, and 34.3% (95% CI: 30.0–38.9) worse (*N* = 452).

### 3.5 Social isolation, insecurity and exposure to violence

Most participants reported that they could rely on relatives or the community for support in case of need (83.3%, 95% CI: 79.8–86.3), while 16.7% (95% CI: 13.7–20.2) declared having none. Regarding social isolation, 51.9% (95% CI: 47.3–56.5) felt surrounded, 39.2% (95% CI: 34.9–43.7) reported isolation or solitude, and 9.0% (95% CI: 6.7–12.0) did not provide a clear answer.

Concerning insecurity, 43.7% of respondents (95% CI: 39.3–48.2) reported feeling unsafe in the gold mining sites.

**Figure 1:**
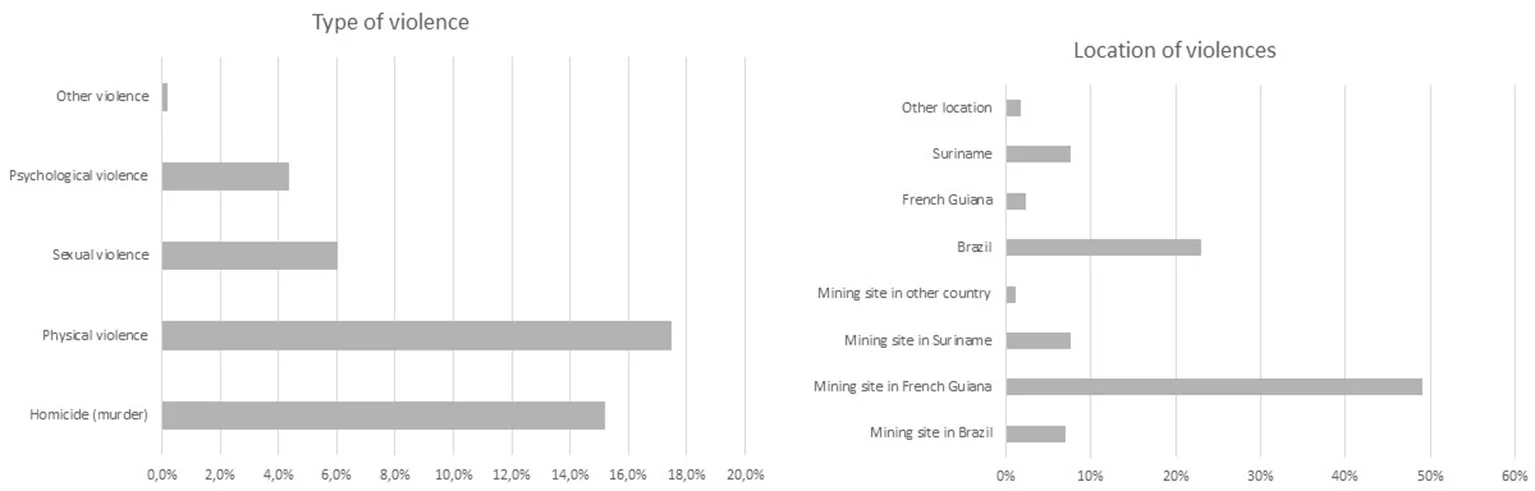
Categories of violence and violence setting reported by gold miners (Orpal4 study, 2024–2025, French Guiana).

Overall, 29.8% of participants (95% CI: 25.7–34.2) reported having been exposed to violence, directly or witnessed, while 68.5% (95% CI: 64.0–72.7) did not, and 1.7% (9 persons) did not wish to answer. This did not differ with gender (33.0% of women, 29.4% of men, *p* = 0.461). Of those 143 persons exposed to violence, most reported it occurred rarely (52.5%), while 26.6% experienced it sometimes and 16.8% often. Type of violence and setting are presented in Figure 1.

### 3.6 Food insecurity

Among the 480 participants, the majority reported not having lacked food in the previous 4 weeks (80.0%), while 19.4% indicated that this had occurred at least occasionally (rarely, sometimes, or often) (0.6% dnk/dnwta). Similarly, 23.8% reported having gone to bed hungry at least once, and 19.2% had experienced a 24-h period without eating.

The global HHS score indicated that 79.0% (95% CI: 75.1–82.4) of participants had no or little hunger, 20.6% (95% CI: 17.2–24.5) suffer from moderate hunger and 0.4% (95% CI: 0.1–1.7) were classified as severe hunger, highlighting a concerning nutritional vulnerability in this population.

The univariate analyses found association between food insecurity and age, country of inclusion, police operation, financial situation, difficulties of movements, isolation, lack of social support and violence exposition. In multivariate model (*N* = 450), food insecurity was associated with age (>40 years: PR = 0.60 [0.43–0.85]), being included in Brazil (PR = 1.56 [1.05–2.32]), having experienced difficulties of movements (PR = 2.02 [1.41–2.88]) being in a difficult financial situation (PR = 1.72 [1.16–2.53]), lacking of social support (PR = 1.52 [1.03–2.23]) and being socially isolated (PR = 1.90 [1.32–2.73]) (no interaction) (Table 2).

**Table 2:** Uni and multivariate analysis of factors associated with food insecurity (Orpal4 study, 2024–2025, French Guiana).

Variable	Category	Food insecurity		Univariate analysis		**Multivariate analysis poisson** ***N*** = **450**	
		Secured (***N*** = **376)** ***n*** **(%)**	**Not secured (*****N*** = **100)** ***n*** **(%)**	**PR** ^*^ **[95% CI]**	***p*** **-value**	**aPR** ^*^ **[95% CI]**	***p*** **-value**
Age (*n* = 476)	< 40 years	157 (74.4)	54 (25.6)	1		1	
	≥40 years	219 (82.6)	46 (17.4)	0.68 [0.48–0.96]	**0.030** ^**^	0.60 [0.43–0.85]	0.004
Sex (*n* = 476)	Female	87 (76,3)	27(23.7)	1		1	
	Male	289 (79.8)	73 (20.2)	0.85 [0.58–1.26]	**0.417**	0.82 [0.55–1.22]	0.323
Country of inclusion (*n* = 476)	Suriname	236 (81.9)	52 (18.1)	1		1	
	Brazil	140 (74.5)	48 (25.5)	1.41 [1.00–2.00]	**0.050**	1.56 [1.05–2.32]	0.028
Time in mining (*n* = 476)	< =10 years	166 (79.4)	43 (20.6)	1			
	>10 years	210 (78.6)	57 (21.4)	1.04 [0.73–1.48]	0.837		
Distance to mining site (*n* = 469)	< 1 day	215 (81.4)	49 (18.6)	1			
	>= 1 day	154 (75.1)	51 (24.9)	1.29 [0.91–1.84]	**0.156**		
Travel cost (*n* = 353)	0–100 Euros	154 (78.6)	42 (21.4)	1			
	>100 Euros	123 (78.3)	34 (21.7)	1.01 [0.68–1.51]	0.959		
Occupation on mine (*n* = 471)	Mobile activity	52 (83.9)	10 (16.1)	1			
	Not mobile activity	321 (78.5)	88 (21.5)	1.33 [0.73–2.43]	0.345		
Police operation (past year) (*n* = 468)	No	201 (83.1)	41 (16.9)	1			
	Yes	169 (74.8)	57 (25.2)	1.47 [1.03–2.09]	**0.034**		
Mouvement difficulties (*n* = 474)	No	268 (84.5)	49 (15.5)	1		1	
	Yes	107 (68.2)	50 (31.8)	2.06 [1.46–2.91]	**< 0.001**	2.02 [1.41–2.88]	< 0.001
Financial situation (*n* = 476)	Comfortable/moderate	288 (81.1)	67 (18.9)	1		1	
	Difficult	58 (69.1)	26 (30.9)	1.64 [1.12–2.41]	**0.012**	1.72 [1.16–2.53]	0.007
	Dnk/dnwta^***^	30 (81.1)	7 (18.9)	1.00 [0.50–2.02]	**0.995**	1.10 [0.50-2.37]	0.827
Quality of life (*n* = 476)	Good	132 (81.0)	31 (19.0)	1			
	Medium	201 (79.8)	51 (20.2)	1.06 [0.71–1.59]	**0.761**		
	Bad/DNK	43 (70.5)	18 (29.5)	1.55 [0.94–2.56]	**0.086**		
Isolation (*n* = 476)	Not isolated	210 (84.3)	39 (15.7)	1		1	
	Isolated	132 (71.0)	54 (29.0)	1.85 [1.29–2.67]	**0.001**	1.90 [1.32–2.73]	0.001
	Don't know/NR	34 (82.9)	7 (17.1)	1.09 [0.52–2.27]	**0.818**	1.29 [0.61-2.74]	0.511
Social support (*n* = 475)	Yes	325 (81.9)	72 (18.1)	1		1	
	No support	50 (64.1)	28 (35.9)	1.98 [1.38–2.85]	**< 0.001**	1.52 [1.03–2.23]	0.033
Perceived insecurity on mining sites (*n* = 471)	No insecurity	160 (78.1)	45 (21.9)	1			
	Insecurity	213 (80.1)	53 (19.9)	1.10 [0.77–1.57]	0.591		
Violence exposition (*n* = 468)	No	267 (81.4)	61 (18.6)	1			
	Yes	103 (73.6)	37 (26.4)	1.42 [0.99–2.03]	**0.054**		

* PR= prevalence ratio/aPR: adjusted prevalence ratio.

** In bold: variables included in the multivariate model.

*** dnk: does not know/dnwta: does not want to answer.

### 3.7 Mental health vulnerability

Overall, 24.2% of participants (95% CI: 20.5–28.2) reported having felt depressed most of the time over the past 2 weeks. Loss of interest or pleasure (anhedonia) was reported by 15.6% (95% CI: 12.6–19.2). 60 people (12.7%, 9.7–15.7) responded “yes” at the 2 questions. In addition, 35.6% (95% CI: 31.3–40.1) reported persistent fatigue or lack of energy. Regarding mental health history, 6.0% of participants (95% CI: 4.2–8.7) reported having been followed or hospitalized by a psychiatrist or psychologist for mental health problems. Exposure to traumatic events was reported by 10.5% (95% CI: 8.0–13.6) of the participants (Supplementary Table 1).

When combining these indicators into a study-specific mental-health vulnerability score, 21.9% (95% CI: 18.2–25.9) of participants were classified as “elevated mental-health vulnerability”.

In univariable analyses, the following factors were associated with a elevated mental-health vulnerability: female sex, financial difficulties, food insecurity, social isolation, exposure to violence, feeling unsafe at mining sites, and having a chronic disease.

In multivariate analysis (*N* = 458), female sex, social isolation and feeling unsafe on the mines were independently associated with elevated mental-health vulnerability: Men had lower odds compared to women (PR = 0.41 [0.29–0.58] *p* < 0.001); isolation was the strongest predictor (PR = 2.24 [1.51–3.34] *p* < 0.001), with insecurity on sites (PR = 2.19 [1.52–3.14]) *p* < 0.001) (Table 3).

**Table 3:** Univariate and multivariate analyses of factors associated with a elevated mental-health vulnerability (Orpal4 study, 2024-2025, French Guiana).

**Variable**	Category	Elevated mental health vulnerability		Univariate analysis		**Multivariate analysis poisson regression** ***N*** = **458**	
		**No** (***N*** = **370)** ***n*** **(%)**	**Yes (*****N*** = **104)** ***n*** **(%)**	**PR** ^*^ **(95% CI)**	***p*** **-value**	**aPR** ^*^ **(95% CI)**	***p*** **-value**
Sex (*N* = 474)	Women	73 (62.9)	43 (37.1)	1		1	
	Men	297 (83.0)	61 (17.0)	0.46 [0.33–0.64]	**< 0.001** ^**^	0.41 [0.29–0.58]	< 0.001
Age (*N* = 474)	≤ 40 years	168 (79.6)	43 (20.4)	1		1	
	>40 years	202 (76.8)	61 (23.2)	1.14 [0.81–1.61]	**0.464**	1.02 [0.73–1.43]	0.893
Country of inclusion (*N* = 474)	Suriname	221 (76.5)	68 (23.5)	1		1	
	Brazil	149 (80,5)	36 (19.5)	0.83 [0.58–1.19]	**0.301**	1.37 [0.92–2.05]	0.126
Police operation in past year (*N* = 466)	No	198 (82.2)	43 (17.8)	1			
	Yes	167 (74.2)	58 (25.8)	1.40 [0.99–1.97]	**0.057**		
Distance to mining site (*N* = 467)	< 1 day	201 (76.7)	61 (23.3)	1			
	≥1 day	163 (79.5)	42 (20.5)	0.88 [0.60–1.22]	0.395		
Financial situation (*N* = 474)	Comfortable/moderate	284 (80.5)	69 (19.5)	1		1	
	Difficult	55 (66.3)	28 (33.7)	1.73 [1.19–2.50]	**0.004**	1.65 [1.12–2.43]	0.011
	Dnk/dnwta^***^	31 (81.6)	7 (18.4)	0.94 [0.47–1.90]	0.869	0.60 [0.28–1.25]	0.174
Mining activity	Mobile	53 (86.9)	8 (13.1)	1			
	Not mobile	313 (76.7)	95 (23.3)	1.78 [0.91–3.47]	**0.093**		
**Time in mining** (N=474)	≤ 10 years	163 (77.6)	47 (22.4)	1			
	>10 years	207 (78.4)	57 (21.6)	0.96 [0.69–1.36]	0.837		
Quality of life (*N* = 474)	Good	132 (81.0)	31 (19.0)	1			
	Medium	196 (78.1)	55 (21.9)	1.15 [0.78–1.71]	0.481		
	Bad/dnk/dnwta	42 (70.0)	18 (30.0)	1.58 [0.96–1.60]	**0.074**		
Food insecurity (*N* = 474)	Secure	301 (80.7)	72 (19.3)	1			
	Insecure	67 (68.4)	31 (31.6)	1.64 [1.15–2.34]	**0.007**		
Social isolation (*N* = 474)	Surrounded	213 (86.6)	33 (13.4)	1		1	
	Isolation/solitude	126 (67.4)	61 (32.6)	2.43 [1.66–3.55]	**< 0.001**	2.24 [1.51–3.34]	0,001
	dnk/dnwta	31 (75.6)	10 (24.4)	1.82 [0.97–3.40]	**0.061**	1.91 [1.00–3.64]	0.050
Social support (*N* = 473)	Yes	310 (78.7)	84 (21.3)	1			
	No support	59 (74.7)	20 (25.3)	1.12 [0.78–1.81]	0.427		
Perceived insecurity on mines(*n* = 471)	No insecurity	229 (86.1)	37 (13.9)	1		1	
	Insecurity	140 (68.0)	66 (32.0)	2.30 [1.61–3.30]	**< 0.001**	2.19 [1.52–3.14]	< 0.001
Violence exposure (*n* = 468)	No	272 (82.9)	56 (17.1)	1			
	Yes	94 (67.1)	46 (32.9)	1.92 [1.37–2.69]	**< 0.001**		
Chronic disease treatment (*N* = 474)	No	345 (79.3)	90 (20.7)	1			
	Yes	25 (64.1)	14 (35.9)	2.30 [1.61–3.30]	**< 0.001**		
Systolic BP ≥160 mmHg (*N* = 474)	< 160 mmHg	354 (78.2)	99 (21.8)	1			
	≥160 mmHg	16 (76.2)	5 (23.8)	1.09 [0.50–2.39]	0.831		

* PR, prevalence ratio; aPR, adjusted prevalence ratio.

** In bold: variables included in the multivariate model.

*** dnk, does not know; dnwta, does not want to answer.

Sensitivity analyses using alternative cut-offs showed broadly consistent directions and magnitudes of association, supporting the robustness of the findings (Supplementary Table 2)

### 3.8 Exploratory analysis

#### 3.8.1 Multiple correspondence analysis (MCA)

The MCA was conducted among 462 participants with complete data. After comparing alternative specifications with different combinations of variables, the final model retained six indicators: mental health vulnerability, food insecurity, exposure to violence, perceived insecurity, social support, and financial difficulties. The first two dimensions were retained because they explained 82.5% of the total inertia (78.4% for Dimension 1 and 4.1% for Dimension 2), thereby providing a concise representation of the main structure of vulnerability in the study population.

- Dimension 1 (global vulnerability gradient): This axis captured the co-occurrence of psychosocial and economic vulnerabilities. The strongest contributors were reporting a high-risk psychological profile (20.7%), exposure to violence (13.9%), perceived insecurity (12.4%), food insecurity (9.6%), and financial difficulties (9.8%), all associated with negative coordinates. This dimension thus represents a continuum ranging from secure and stable conditions (positive coordinates) to cumulative vulnerability (negative coordinates), where individuals simultaneously experience economic hardship, lack of support, and exposure to violence.
- Dimension 2 (type of vulnerability: social vs. contextual): The main contributors were lack of social support (26.9%), food insecurity (17.9%), and financial difficulties (10.2%), opposed to perceived insecurity (10.4%) and exposure to violence (7.3%) on the negative side. This secondary axis differentiates social and financial isolation from contextual insecurity, suggesting distinct yet complementary facets of vulnerability within the study population.

#### 3.8.2 Cluster analysis

**Figure 2:**
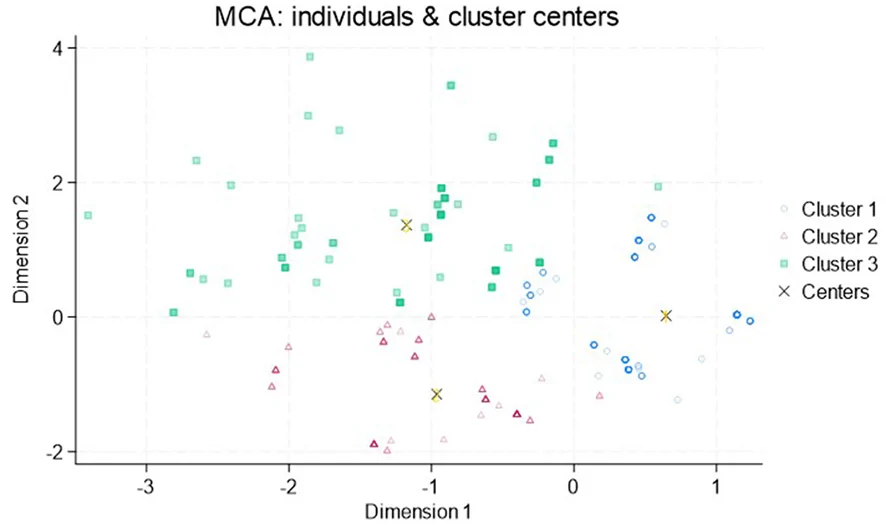
Multiple correspondence analysis (MCA) and k-means clustering (*k* = 3) based on six vulnerability indicators (Orpal4 study, 2024–2025, French Guiana).

The MCA-based clustering identified three distinct vulnerability profiles among the 462 participants included in the analysis (Figure 2 and Table 4).

**Table 4:** Distribution of vulnerability indicators across the three clusters (Orpal4 study, 2024–2025, French Guiana).

**Cluster**	*n*	High-risk profile	Perceived insecurity	Exposure to violence	Food insecurity	Lack of social support	Financial difficulties
Cluster 1 – globally secure and supported	209	6.2%	24.9%	12.0%	0%	0%	19.1%
Cluster 2 – contextually exposed but socially supported	107	58.9%	94.4%	76.6%	16.8%	11.2%	32.7%
Cluster 3 – socioeconomically vulnerable	146	16.4%	43.3%	21.2%	53.4%	16.4%	33.1%

- Cluster 1: Globally secure and supported (*n* = 209, 45.2%) showed the lowest prevalence of all vulnerability indicators, including high-risk psychological profile (6.2%), exposure to violence (12.0%), perceived insecurity (24.9%), food insecurity (0%), lack of social support (0%), and financial difficulties (19.1%).
- Cluster 2: Contextually exposed but socially supported (*n* = 107, 23.2%) was characterized by high perceived insecurity (94.4%), frequent exposure to violence (76.6%), and a high-risk psychological profile (58.9%). However, the prevalence of food insecurity (16.8%), financial difficulties (32.7%), and lack of social support (11.2%) was comparatively low.
- Cluster 3: Socioeconomically vulnerable (*n* = 146, 31.6%) was mainly characterized by food insecurity (53.4%) and financial difficulties (53.4%), together with a higher prevalence of lack of social support (43.8%). Perceived insecurity (32.2%), exposure to violence (21.2%), and psychological risk (16.4%) were less frequent than in Cluster 1.

These profiles illustrate distinct but complementary forms of vulnerability: contextual insecurity vs. socio-economic deprivation. Given the exploratory nature of this analysis, these clusters should be interpreted as descriptive patterns rather than definitive or clinically validated subgroups.

#### 3.8.3 Sociodemographic and health characteristics across clusters

We then compared sociodemographic and health characteristics across the three clusters (Table 5). Most characteristics did not differ significantly between clusters. However, forgone care was more frequent in Cluster 2 (19.6%) than in Clusters 1 (8.7%) and 3 (11.0%; *p* = 0.016). Participants in Cluster 2 were also more likely to report that their current life was worse than before (46.5 vs. 25.6% in Cluster 1 and 40.1% in Cluster 3; *p* = 0.004) and to report police operations during the previous year (57.0 vs. 41.6% and 50.7%; *p* = 0.026). Smartphone ownership was highest in Cluster 1 (97.1 vs. 88.8% and 91.8%; *p* = 0.010).

**Table 5:** Sociodemographic and health characteristics of participants by cluster (Orpal4 study, 2024–2025, French Guiana).

Characteristic	Cluster 1: Globally secure and supported *N* = 209	Cluster 2: Contextually exposed but socially supported *N* = 107	Cluster 3: Socioeconomically vulnerable *N* = 146	*p*-value
Sex: women	45 (21.5%)	31 (29.0%)	36 (24.7%)	0.341
Age >40 years	109 (52.2%)	63 (58.9%)	82 (56.2%)	0.493
Low education	133 (63.6%)	69 (64.5%)	88 (60.3%)	0.744
Country: Brazil	85 (40.7%)	38 (35.5%)	55 (37.7%)	0.650
>10 years in mining	112 (53.6%)	57 (53.3%)	86 (58.9%)	0.551
Mobile occupation	32 (15.5%)	12 (11.3%)	16 (11.1%)	0.406
Distance to site ≥1 day	84 (41.8%)	38 (37.3%)	72 (50.3%)	0.101
Medium/bad perceived health	105 (50.2%)	66 (61.7%)	76 (52.1%)	0.143
Desisted from care	18 (8.7%)	21 (19.6%)	16 (11.0%)	**0.016**
Chronic disease	14 (6.7%)	9 (8.4%)	13 (8.9%)	0.720
Systolic BP ≥160 mmHg	6 (2.9%)	4 (3.7%)	8 (5.5%)	0.456
Poor quality of life	22 (10.5%)	16 (15.0%)	20 (13.7%)	0.152
Life worse than before	51 (25.6%)	47 (46.5%)	55 (40.1%)	**0.004**
Police operation in the previous year	87 (41.6%)	61 (57.0%)	74 (50.7%)	**0.026**
Smartphone ownership	203 (97.1%)	95 (88.8%)	134 (91.8%)	**0.010**
Internet connection	183 (87.6%)	90 (84.1%)	124 (84.9%)	0.647

In bold are the significant results.

## 4 Discussion

### 4.1 Main results

Our study sheds light on a set of less visible but critical determinants of health affecting clandestine gold miners in French Guiana. More than half of participants (52.7%) rated their health as medium or poor, and nearly half lived more than one day of travel—by foot or boat—from the recruitment place. Social vulnerability was substantial: 16.7% (13.7–20.2) reported having no social support, and 43.7% (39.3–48.2) felt unsafe at mining sites. Food insecurity was also prominent, with 20.5% (17.2–24.5) experiencing moderate hunger and 0.4% (0.1–1.7) classified as severely food insecure. Based on a composite psychological risk score, 21.9% (18.2–25.9) of miners were considered at elevated mental-health vulnerability. Food insecurity was associated with inclusion in Brazil (OR = 1.87), financial difficulties (OR = 2.12), lack of social support (OR = 1.88), and social isolation (OR = 2.56). Mental-health vulnerability was associated with female sex (PR = 0.27 for men), social isolation (PR = 2.63), and feeling unsafe at mining sites (PR = 2.63). Cluster analysis identified three vulnerability profiles: a globally secure and supported cluster (*n* = 295, 61%), a highly exposed but supported cluster (*n* = 106, 22%), and an isolated and financially deprived cluster (*n* = 61, 17%).

### 4.2 Limitations

Although the target sample size, calculated based on malaria prevalence, was not reached because fewer potentially eligible individuals were available at the recruitment sites than anticipated, the precision of the vulnerability estimates remained acceptable, with reasonably narrow confidence intervals. Self-reported data may be affected by recall and social desirability biases. Underreporting of sensitive issues (violence, mental health) was likely, though efforts were made to ensure confidentiality – including the use of tarpaulin partitions and distanced seating – complete privacy could not be guaranteed. Recruiting participants at locations where they go when police operation occur on the mines may have biased reported timing since the last operation. Screening tools have limitations: PHQ-2 was used in a binary way and our composite vulnerability score was exploratory and unvalidated. Clustering analysis was exploratory; group characteristics varied slightly across model specifications, though a consistent gradient from low to high vulnerability emerged. The cross-sectional design was appropriate for describing the situation and exploring associations, but it precluded establishing temporal or causal relationships; moreover, convenience and snowball sampling at logistical bases may have excluded the most isolated, severely ill, or less mobile miners, thereby limiting the generalizability of the findings to all informal gold miners in French Guiana.

### 4.3 Specific drivers of food insecurity in gold mining population

Food supply in highly isolated mining sites is structurally complex. Although miners frequently resort to hunting and fishing, most of their time is devoted to gold mining activities (4, 9). Food provision—usually managed by the camp leader—relies on complex logistical circuits designed to bypass police checkpoints, resulting in limited availability of fresh products. High mobility further hampers food production, as miners do not engage in cultivation (4). Although the majority of participants reported little or no hunger, one in five experienced food insecurity. These levels were notably lower than those observed in vulnerable urban coastal neighborhoods in French Guiana during the COVID-19 pandemic, where Basurko et al. reported 23.8% severe hunger and 42.6% moderate hunger using the same Household Hunger Scale (HHS) (21). However, the HHS captures only severe food insecurity and may therefore underestimate the overall burden. Beyond quantitative food insufficiency, dietary diversity remains a major concern, although it was not assessed in the present study. Nutritional deficiencies have previously affected this population, as illustrated by reported beriberi epidemics—a vitamin B1 deficiency—leading to cases of fatal cardiac failure among gold miners (22). Several factors were found to be associated with food insecurity. First, food insecurity was associated with police operations in univariate analyses, although this relationship did not persist in multivariate models. First, according to interviewees, police operations targeting logistical infrastructure have sometimes involved the destruction of food supplies and medicines. Such reports suggest that these operations may disrupt access to essential goods in remote sites, although this association was not confirmed in multivariate analyses. Second, many miners originate from Brazil's North and Northeast regions—particularly Maranhão—where baseline food insecurity is substantially higher than in southern Brazil (23). Although direct prevalence comparisons are limited due to the use of different assessment tools (EBIA vs. HHS), this suggests that migration to French Guiana does not fully achieve its intended goal of improved living conditions, even though 48% of participants reported having a better life than in Brazil. Finally, food insecurity was strongly associated with lack of social support and isolation, as previously reported in other studies although the cross-sectional design prevents determining the causality of these relationships (24). In the cluster analysis, the most precarious subgroup was characterized by food insecurity, poor self-reported health, recent exposure to police operations, and lack of smartphone ownership. These findings underscore the protective role of social capital and network integration against material deprivation in clandestine mining contexts.

### 4.4 Mental health: a high burden

Overall, 21.9% of participants were classified as having an elevated mental-health vulnerability, and 12.7% responded “yes” to the binary PHQ-2 screening items, indicating that they would have required further evaluation for depression. This prevalence remains difficult to compare with other populations, as the questions used in our survey were not standardized. In French Guiana, a 2024 general-population study reported that 14% of adults aged 18–79 years had experienced a major depressive episode in the previous 12 months, yet only 29% sought professional care. In the same study, 6% had suffered from generalized anxiety disorder over the past 12 months, and 64% of them did not access any health services (25). In Brazil, the proportion of adults aged 18 years or older who had ever received a diagnosis of depression from a mental health professional was estimated at 10.2% (compared with 7.6% in 2013) (23). According to the Pan American Health Organization, Brazil has the second highest level of age-standardized DALYs for mental disorders per 100,000 population after the USA (26). Few studies described psychological distress in other mining contexts in Latin America. Among informal miners in northern Chile, the reported prevalence estimates ranged from 6 to 28.5%, depending on the cut-off applied to the GHQ-12 scale (27). Although differences in measurement instruments and study designs limit direct comparisons, our results point to a considerable mental health burden in a population facing chronic stressors, social isolation, and repeated exposure to violence. Evidence from studies in Ghana highlights strong links between depression, social support, and food insecurity (28, 29). The fact that such associations remained significant only in univariate analyses in our study underscores the multifactorial and highly interdependent nature of vulnerability mechanisms in this setting.

### 4.5 Exposure to violence: a defining feature of clandestine mining

Violence was highly prevalent in our sample: 43.7% (95% CI: 39.3–48.2) reported feeling unsafe at mining sites, and 29.8% reported direct or witnessed exposure to physical violence. Importantly, when asked where violence occurred, 49% indicated “in gold mining sites in French Guiana”, followed by 23% in Brazil. These findings must be understood within the broader context of violence in the region. In Brazil, assaults account for 29.8% of deaths among 20–29-year-olds, and homicide is the leading cause of death for those aged 15–29 (30). “Legal interventions and war operations” contribute to 3.1% of deaths among 15–19-year-olds and 2.2% among 20–29-year-olds. Compared to the general Brazilian population—where 18.3% of adults report experiencing violence (30)—the violence profile among miners in French Guiana appears distinct. In Brazil, psychological violence predominates (17.4%), followed by physical (4.1%) and sexual violence (0.8%). In French Guiana, however, physical and sexual violence were more prominent. This difference may partly reflect the hazardous nature of mining activities and the predominantly male composition of the mining workforce. Moreover, 15% of our sample reported witnessing homicide, an extreme form of traumatic exposure with lasting psychological consequences. The concentration of reported violence on mining sites in French Guiana indicates that the clandestine mining sector itself generates significant interpersonal violence, which might be compounded by criminalized status, high alcohol consumption, and lack of legal protection (9, 31). By comparison, workplace violence among formal miners in Chile (18%) was lower than in our sample (43.7%), likely reflecting differences in legal status, labor protections, and site governance (27). Although post-traumatic stress disorder (PTSD) could not be assessed in the present study, a high prevalence may reasonably be anticipated in this population, given the high levels of exposure to violence among illegal gold miners. This assumption is further supported by the already high prevalence of PTSD reported in the adult population of coastal French Guiana (7.6%), nearly 10 times that observed in mainland France (32).

### 4.6 Social isolation and limited access to care

Lack of social support was reported by 16.7% (95% CI: 13.7–20.2) of participants. It seems lower than the 24% reporting low support in Chilean miners but the measure is different (27). Isolation is compounded by extreme geographic remoteness. Most participants reported that the nearest health center was in Maripasoula, requiring an average of 3 days of travel (range: 1–15 days), with a prohibitive estimated cost (210–700 USD). This physical and financial inaccessibility to care likely exacerbates untreated medical and mental health conditions. Self-reported health status was rated as good by 46.5%, medium by 46.9%, and poor by 5.8%, of our population which was slightly worse than the Brazilian population where 66.1% report good or very good health, 28.1% fair, and 5.8% poor (23).

### 4.7 A multidimensional and heterogeneous vulnerability shaped by external factors

The multidimensional vulnerabilities experienced by clandestine gold miners in French Guiana differ markedly from those observed both in others populations of French Guiana and among mining populations elsewhere in Latin America. Within the mining population itself, distinct vulnerability patterns emerge, including a globally secure and supported group (61%), a moderately vulnerable group (23%), and a highly vulnerable group (16%). The extent to which vulnerability is exacerbated by the criminalization of this population—composed of undocumented migrants engaged in an illegal activity—warrants critical examination. Criminalization may extend beyond legal frameworks to include processes of social stigmatization and public moral judgment (33–35). In French Guiana, clandestine gold miners are widely perceived negatively by the general population, often portrayed as “stealing” local gold, destroying the rainforest, and polluting the environment. As a result, they are commonly viewed as a threat to public order rather than as individuals exposed to an inequitable social order. However, our data indicate that migration is frequently considered by these individuals as the only viable option in the context of socio-economic precarity in their countries of origin. This situation comes at a substantial cost to their physical and mental health, as documented in numerous previous studies (5, 6, 9). Their marginalization thus stems from broader structural dynamics that extend beyond individual behaviors, ultimately undermining their dignity and humanity.

## 5 Conclusion

While initial research efforts have primarily focused on malaria, other infectious diseases and chronic conditions, the present study broadens the scope of health issues affecting clandestine gold miners. Our findings highlight the major role of psychosocial determinants—particularly food insecurity, mental health vulnerability, exposure to violence, and social isolation—which remain largely unaddressed. Repressive approaches exacerbate mechanisms of structural exclusion, thereby increasing vulnerability. Rehumanizing this marginalized population should be considered an important policy objective. Possible responses may include harm reduction strategies that rely on community-based health services and improved dissemination of information on access to care. Health professionals could be informed and trained to identify and account for their specific vulnerabilities. At a broader level, socio-economic development in the regions of origin of gold miners could improve living conditions and reduce the motivation for migration, which remains driven by the hope of a better life. Strengthened cross-border cooperation with Brazil and Suriname could be considered to improve access to care and address the socioeconomic factors affecting mobile and marginalized populations.

## Data Availability

The datasets presented in this article and the datasets generated during the current study are not readily available because the study population is particularly vulnerable and unrestricted data sharing could pose risks to participants' privacy and safety. However, de-identified data may be made available from the corresponding author upon reasonable request and subject to controlled access, ethical approval where applicable, and appropriate data-use agreements. Requests to access the datasets should be directed to Maylis Douine, maylis.douine@chu-guyane.fr.
