# Supplementary material for: Psychosocial vulnerability of people working in informal gold mines in French Guiana: a multicentric cross-sectional survey in 2024–2025
Source: Front Public Health. 2026 Sep 11;14:1894124. doi: 10.3389/fpubh.2026.1894124 (PMC13612180; doi:10.3389/fpubh.2026.1894124)
Supplement: Supplementary file 1 [file Table_1.docx]

Supplementary Material

# 1 Supplementary material 1 : Observation Booklet

Study Title

Radical Cure for Malaria Among Highly Mobile and Hard-to-Reach Populations in the Guiana Shield

Post Intervention Survey

Short Title

ORPAL_4

**Eligibility Criteria**

-	Do you work in gold mining in French Guiana?

• Yes

• No

-	Did you leave the mining site less than 7 days ago?

• Yes

• No

-	Are you over 18 years old?

• Yes

• No

-	Is this your first participation in the ORPAL_4 study?

• Yes

• No

-	Signed written informed consent:

• Yes

• No

-	Inclusion date: … / … / …

Initials of the investigator: …

Inclusion site: …

Participant number

**Sociodemographic Data**

-	Gender:

• Female

• Male

• Other (specify)

-	If female: Are you pregnant?

• Yes – How many months? …

• No

• Prefers not to answer

• Does not know

-	Age (in years): …

-	Country of birth:

• Brazil

• French Guiana

• Suriname

• Guyana

• Dominican Republic

• Other country (specify)

• Prefers not to answer

• Does not know

-	If born in Brazil: State of birth:

• Amapá

• Pará

• Roraima

• Maranhão

• Other state (specify)

• Prefers not to answer

• Does not know

-	Level of schooling:

• None

• Primary education (1–4 years of school completed)

• Secondary education (5–12 years completed)

• Higher education (university, college, etc.)

• Prefers not to answer

• Does not know

**Gold Mining Activity**

How long have you worked in gold mining in total?

Indicate duration:

• … days

• … weeks

• … months

• … years

• Prefers not to answer

• Does not know

In which mining site (“garimpo” or “grota”) do you currently work?

Provide as much detail as possible and mention both names if different.

• Free text (two lines)

• Prefers not to answer

• Does not know

Do you know the name of the river and/or the community closest to the mining site where you currently work?

• Yes

 • Name of river: …

 • Name of community: …

• No

• Prefers not to answer

• Does not know

How long have you been at this mining site?

Indicate duration:

• … days

• … weeks

• … months

• … years

• Prefers not to answer

• Does not know

What is the distance from this mining site, and how long did your trip take to get here (relative to the inclusion site)?

• … hours

• … days

• Prefers not to answer

• Does not know

How much did you pay for the trip from the mining site to here?

Amount paid: …

Currency:

• EUR

• SRD

• BRL

• USD

• Gram of gold

• Other (specify)

What is your current occupation at the mining site?

(Multiple answers allowed)

• Chainsaw operator

• Machine operator

• Sex worker

• Boatman / canoe operator

• Transporter / carrier / fuel carrier

• Quad bike driver

• Bar or cabaret worker

• Machine owner

• Mining site manager

• Bar or cabaret owner

• Shop or sales worker

• Shop owner

• Cleaner / cook

• Street vendor

• Prospector

• "Piupiuzeiro"

• Miner

• Unemployed

• Other (specify)

• Prefers not to answer

• Does not know

What type of mining site do you currently work in, and how is gold extracted there?

(Multiple answers allowed)

• “Baranco”

• Pit / shaft (“poço”, “pui”)

• Open‑air mining

• Raft (“balsa”)

• Other (specify)

• Not applicable (for mobile professions such as street vendors)

• Prefers not to answer

• Does not know

Why did you come out of the mining site this time?

(Multiple answers allowed)

• Rest

• Visit family

• Medical treatment

• Purchase of work materials

• Purchase of food

• Personal shopping

• Police / gendarmerie / army operation

• Other reason (specify)

• Prefers not to answer

• Does not know

How long do you expect to stay here (including days already spent)?

• … days

• … weeks

• … months

• … years

• All the time (with occasional trips back to the mining site)

• Prefers not to answer

• Does not know

When was the last time you left the mining site before this current exit?

• … days ago

• … weeks ago

• … months ago

• … years ago

• This is my first time leaving the mining site

• Prefers not to answer

• Does not know

In which countries have you worked in gold mining over the past 3 years?

(Multiple entries allowed)

• Number of mining sites in French Guiana: …

• Number of mining sites in Brazil: …

 • In which Brazilian states? …

• Number of mining sites in Suriname: …

• Other country (specify): …

 • Number of mining sites in that country: …

In the past 2 years, have you encountered unusual or new difficulties when traveling between mining sites or when leaving a mining site?

• Yes

• No

• Prefers not to answer

• Does not know

If yes, for what reasons? (Multiple answers allowed)

• Because of police

 • Where? …

• Because of drought

• Other reason (specify)

**Gold Mining Determinants**

Why did you start gold mining?

• Free text

Why do you continue working in gold mining?

• Free text

Have you experienced a police operation at your mining site during the past year?

• Yes

• No

• Prefers not to answer

• Does not know

If yes:

How many times?

• Free text

When was the most recent one?

• … days

• … weeks

• … months

• … years

Communication Channels

Do you have a smartphone?

• Yes

• No

• Prefers not to answer

• Does not know

If yes: what operating system?

• iOS (iPhone)

• Android

• Other (specify)

• Prefers not to answer

• Does not know

During your last stay in the mining site, did you have internet access?

• Yes

• No

• Prefers not to answer

• Does not know

**Quality of Life in the Mining Sites**

How would you rate your quality of life?

• Poor

• Neither good nor bad

• Good

• Prefers not to answer

How is your quality of life in mining sites compared to your home country?

• Better

• The same

• Worse

• Does not know

How would you describe your current financial situation?

• Difficult

• Moderate

• Comfortable

• Not a concern

• Prefers not to answer

• Does not know

**Food Insecurity**

In the past 4 weeks (30 days), was there ever no food available because you could not afford it or because food was not available in the mining site?

• Never

• Rarely (1–2 times)

• Sometimes (3–10 times)

• Frequently (more than 10 times)

• Prefers not to answer

• Does not know

In the past 4 weeks, did you go to bed hungry at night due to lack of food?

• Never

• Rarely (1–2 times)

• Sometimes (3–10 times)

• Frequently (more than 10 times)

• Prefers not to answer

• Does not know

In the past 4 weeks, did you spend a whole day and night without eating because of lack of food?

• Never

• Rarely (1–2 times)

• Sometimes (3–10 times)

• Frequently (more than 10 times)

• Prefers not to answer

• Does not know

**Isolation and Social Life**

If you were in need, do you think you could rely on someone (household member, family, friends, neighbours, colleagues, community) for moral or emotional support?

• Yes

• No

• Prefers not to answer

• Does not know

In general, would you say you feel:

• Very lonely

• A little lonely

• With a sufficient support network

• With an excellent support network

• Prefers not to answer

• Does not know

**Access to Healthcare**

In the last 12 months, did you give up any healthcare or medical service?

• Yes

• No

• Prefers not to answer

If yes, why? (Multiple answers allowed):

• Very long waiting time

• Difficulty scheduling appointments / inconvenient opening hours

• Not enough doctors

• Cannot afford a translator

• Financial reasons

• Too far / access difficulties

• Difficulties accessing healthcare coverage

• Problems with identity documents

• Dislike of going to the doctor

• Fear of not being understood

• Other (specify)

• Prefers not to answer

• Does not know

**Clinical Data**

How would you describe your health?

• Good

• Average

• Poor

• Prefers not to answer

• Does not know

Are you currently on long‑term or chronic treatment?

• Yes

• No

• Prefers not to answer

• Does not know

If yes, for what reason? (Multiple answers allowed)

• High blood pressure

• Diabetes

• Gastritis or ulcer

• Asthma (including allergic asthma)

• Chronic bronchitis / COPD / emphysema

• Heart disease

• Stroke or chronic consequences of stroke

• Osteoarthritis (excluding arthritis)

• Lower back or spinal chronic problem

• Liver cirrhosis

• Depression

• Dyslipidemia

• Cancer – type: …

• Other (specify)

• Prefers not to answer

• Does not know

During the last week, did you take your daily treatment every day?

• Yes

• No

• I do not remember

• Prefers not to answer

• Does not know

If no, why?

• Treatment interruption

• Too far from a health center or hospital

• Too expensive

• Other reason (specify)

• Prefers not to answer

• Does not know

**Violence Exposure**

Do you know someone (or you) who has been a victim of violence?

• Yes

• No

• Prefers not to answer

If yes: What type of violence? (Multiple answers allowed)

• Murder

• Sexual violence

• Physical violence

• Psychological violence

• Other (specify)

If yes: Where did the violence occur? (Multiple answers allowed)

• Mining site in Brazil

• Mining site in French Guiana

• Mining site in Suriname

• Mining site elsewhere

• In Brazil

• In French Guiana

• In Suriname

• In another place

If yes: How often?

• Rarely (1–2 times)

• Sometimes (1–2 times)

• Frequently (more than 10 times)

Do you currently feel safe where you live in the mining site?

• Yes

• No

• Prefers not to answer

• Does not know

**Anxiety / Depression**

In the past two weeks, have you felt sad or depressed most of the day, nearly every day?

• Yes

• No

• Prefers not to answer

• Does not know

In the past two weeks, have you lost interest or pleasure in activities you usually enjoy?

• Yes

• No

• Prefers not to answer

• Does not know

In the past two weeks, have you felt tired or low‑energy almost all the time?

• Yes

• No

• Prefers not to answer

• Does not know

Have you ever been treated or hospitalized by a psychiatrist or psychologist for mental health problems?

• Yes

• No

• Prefers not to answer

Do you think you have experienced one or more traumatic events in your life?

• Yes

• No

• Prefers not to answer

Supplementary Material should be uploaded separately on submission. Please include any supplementary data, figures and/or tables.

Supplementary material is not typeset so please ensure that all information is clearly presented, the appropriate caption is included in the file and not in the manuscript, and that the style conforms to the rest of the article.

# 2 Supplementary Figures and Tables

## 2.1 Supplementary Table 1: Mental Health evaluation by the participants:

| Question | Response | n | % | 95% CI |
| --- | --- | --- | --- | --- |
| During the past 2 weeks, have you felt sad, gloomy, depressed, most of the time during the day and almost every day? | Yes | 116 | 24.2 | 20.5–28.2 |
|  | No | 360 | 75.0 | 70.8–79.0 |
|  | Don’t know / No answer | 4 | 0.8 | 0.3–2.2 |
| During the past 2 weeks, do you almost always feel you don’t like anything, you’ve lost interest or pleasure in the things you usually enjoy? | Yes | 75 | 15.6 | 12.6–19.2 |
|  | No | 399 | 83.1 | 79.4–86.1 |
|  | Don’t know / No answer | 6 | 1.2 | 0.5–2.8 |
| During the past 2 weeks, did you feel tired almost all the time, without energy? | Yes | 171 | 35.6 | 31.3–40.1 |
|  | No | 305 | 63.5 | 59.0–67.8 |
|  | Don’t know / No answer | 4 | 0.8 | 0.3–2.2 |
| Have you ever been followed or hospitalized by a psychiatrist or psychologist for mental health problems? | Yes | 29 | 6.0 | 4.2–8.7 |
|  | No | 448 | 93.3 | 91.0–95.4 |
|  | No answer | 3 | 0.6 | 0.2–1.9 |

## 2.2 Supplementary Table 2: Sensitivity analyses using alternative cut-offs for the study-specific mental-health vulnerability score (In bold are highlighted the significant associations)

|  |  | **Mental Health Vulnerability Score** | | |
| --- | --- | --- | --- | --- |
| **Variable** | **Category** | **0 vs 1–4** | **0–1 vs 2–4** | **0–2 vs 3–4** |
|  | n/N prevalence % [CI95%] | 215/474 45.4 [40.8, 50.0] | 104/474 21.9 [18.3, 25.9] | 64/474 13.5 [10.6, 16.9] |
| sex | women | 1 | 1 | 1 |
|  | men | OR 0.54 [0.35, 0.82]; 0.00 | OR 0.35 [0.22, 0.56]; 0.00 | OR 0.35 [0.20, 0.61]; 0.00 |
| age | ≤40 years | 1 | 1 | 1 |
|  | >40 years | OR 1.31 [0.91, 1.88]; 0.15 | OR 1.18 [0.76, 1.83]; 0.46 | OR 1.12 [0.65, 1.90]; 0.69 |
| country of inclusion | Suriname | 1 | 1 | 1 |
|  | Brasil | **OR 0.70 [0.48, 1.02]; 0.06** | OR 0.79 [0.50, 1.24]; 0.30 | OR 0.67 [0.38, 1.19]; 0.17 |
| police operation last year | no | 1 | 1 | 1 |
|  | yes | OR 1.27 [0.88, 1.82]; 0.20 | **OR 1.53 [0.99, 2.37]; 0.06** | **OR 1.74 [1.02, 2.98]; 0.04** |
| number of police operation last year | none | 1 | 1 | 1 |
|  | <=5 | OR 1.43 [0.94, 2.16]; 0.09 | **OR 1.98 [1.23, 3.20]; 0.01** | **OR 2.01 [1.12, 3.61]; 0.02** |
|  | >5 | OR 1.03 [0.62, 1.70]; 0.92 | OR 0.84 [0.43, 1.66]; 0.62 | OR 1.21 [0.56, 2.63]; 0.63 |
| distance mine-inclusion site | < 1 day | 1 | 1 | 1 |
|  | >=1 day | OR 0.73 [0.51, 1.07]; 0.11 | OR 0.82 [0.52, 1.29]; 0.39 | OR 0.80 [0.46, 1.39]; 0.43 |
| financial situation | comfortable or moderate | 1 | 1 | 1 |
|  | difficult | **OR 1.79 [1.10, 2.90]; 0.02** | **OR 2.10 [1.24, 3.54]; 0.01** | **OR 2.26 [1.23, 4.14]; 0.01** |
|  | dnk/dnwta | OR 1.37 [0.70, 2.68]; 0.36 | OR 0.93 [0.39, 2.20]; 0.87 | OR 0.90 [0.30, 2.65]; 0.84 |
| occupation on mine | mobile activity | 1 | 1 | 1 |
|  | not mobile activity | **OR 2.57 [1.41, 4.70]; 0.00** | OR 2.01 [0.92, 4.38]; 0.08 | **OR 3.33 [1.01, 10.98]; 0.05** |
| time in mining | <=10 years | 1 | 1 | 1 |
|  | >10 years | OR 0.97 [0.68, 1.40]; 0.89 | OR 0.95 [0.62, 1.48]; 0.84 | OR 1.19 [0.70, 2.03]; 0.52 |
| quality of life | good | 1 | 1 | 1 |
|  | medium | OR 1.21 [0.81, 1.81]; 0.34 | OR 1.19 [0.73, 1.96]; 0.48 | OR 1.35 [0.74, 2.47]; 0.33 |
|  | bad/dnk/dnwta | OR 1.75 [0.96, 3.18]; 0.07 | OR 1.82 [0.93, 3.59]; 0.08 | OR 1.61 [0.70, 3.72]; 0.26 |
| isolation | not isolated | 1 | 1 | 1 |
|  | isolated | **OR 2.33 [1.58, 3.44]; 0.00** | **OR 3.12 [1.94, 5.04]; 0.00** | **OR 3.01 [1.71, 5.30]; 0.00** |
|  | dnk/dnwta | OR 1.09 [0.55, 2.15]; 0.80 | OR 2.08 [0.93, 4.64]; 0.07 | OR 0.55 [0.12, 2.44]; 0.43 |
| social support | yes | 1 | 1 | 1 |
|  | No support | OR 1.07 [0.66, 1.74]; 0.79 | OR 1.25 [0.71, 2.19]; 0.43 | OR 1.48 [0.77, 2.84]; 0.23 |
| violence exposition | no | 1 | 1 | 1 |
|  | yes | **OR 2.37 [1.58, 3.56]; 0.00** | **OR 2.38 [1.51, 3.75]; 0.00** | **OR 1.79 [1.04, 3.10]; 0.04** |
| food insecurity | secure | 1 | 1 | 1 |
|  | insecure | **OR 1.57 [1.00, 2.45]; 0.05** | **OR 1.93 [1.18, 3.18]; 0.01** | **OR 2.55 [1.44, 4.52]; 0.00** |
| insecurity | no | 1 | 1 | 1 |
|  | yes | **OR 2.29 [1.58, 3.32]; 0.00** | **OR 2.92 [1.85, 4.59]; 0.00** | **OR 2.84 [1.63, 4.94]; 0.00** |
| chronic disease | no | 1 | 1 | 1 |
|  | yes | **OR 2.30 [1.17, 4.55]; 0.02** | **OR 2.15 [1.07, 4.30]; 0.03** | OR 2.07 [0.93, 4.60]; 0.07 |
| high blood pressure | <160 mmHg | 1 | 1 | 1 |
|  | ≥160 mmHg | OR 1.64 [0.68, 3.97]; 0.27 | OR 1.12 [0.40, 3.13]; 0.83 | OR 1.54 [0.50, 4.74]; 0.45 |
|  | |  |  |  |

# 3 Supplementary Figure 1 : Radar Plot of socio-demographic and health characteristics across the three identified clusters.
